# Comparative Analysis of Normalization Methods for Network Propagation

**DOI:** 10.3389/fgene.2019.00004

**Published:** 2019-01-22

**Authors:** Hadas Biran, Martin Kupiec, Roded Sharan

**Affiliations:** ^1^School of Electrical Engineering, Tel Aviv University, Tel Aviv, Israel; ^2^School of Molecular Cell Biology and Biotechnology, Tel Aviv University, Tel Aviv, Israel; ^3^Blavatnik School of Computer Science, Tel Aviv University, Tel Aviv, Israel

**Keywords:** network diffusion, protein–protein interaction network, gene prioritization, *p*-value computation, degree-preserving randomization, telomere length maintenance

## Abstract

Network propagation is a central tool in biological research. While a number of variants and normalizations have been proposed for this method, each has its own shortcomings and no large scale assessment of those variants is available. Here we propose a novel normalization method for network propagation that is based on evaluating the propagation results against those obtained on randomized networks that preserve node degrees. In this way, our method overcomes potential biases of previous methods. We evaluate its performance on multiple large scale datasets and find that it compares favorably to previous approaches in diverse gene prioritization tasks. We further demonstrate its utility on a focused dataset of telomere length maintenance in yeast. The normalization method is available at http://anat.cs.tau.ac.il/WebPropagate.

## Introduction

Network propagation is a method of choice for diverse analyses such as protein function prediction, gene prioritization and identification of disease modules (Cowen et al., 2017). There are at least 17 available software tools that employ different variants of network propagation for these purposes (Cowen et al., 2017; Biran et al., 2018).

However, the basic propagation technique has some known limitations: First, raw propagation scores do not carry any statistical significance information and can only be used to rank proteins. Second, they are greatly affected by the degrees of initial proteins implicated in the process under study (termed seed set below) and the degree of any candidate protein being scored. This biases the results toward high degree, well studied proteins.

To deal with the second challenge, Erten et al. (2011) suggested the DADA normalization approach. This method normalizes the raw propagation scores with the eigenvector centrality measure for each protein, and then produces ranks based on either these normalizations or the raw propagation scores, depending on the seed set average weighted degree.

Mazza et al. (2016) tackled the first challenge by evaluating propagation scores against those obtained from propagating random seed sets. Nevertheless, none of the methods solves both problems, calling for a more complete solution.

In this work we present a novel normalization technique that tackles both challenges. We developed a new technique, in which the raw propagation scores are normalized through propagation scores obtained in random degree-preserving networks (RDPN). In cross validation tests, our method outperforms previous normalizations in gene prioritization tasks on diverse disease-related and function-related data sets in both human and yeast. Furthermore, it eliminates the degree biases of previous approaches and allows the assessment of statistical significance of the results by providing *p*-values that are corrected for multiple testing of candidate proteins.

## Results

### Network Propagation

Network propagation is a process in which a preselected set of seed proteins that underlie some phenotype of interest are viewed as “heat sources” in a PPI network. The heat is diffused to the rest of the proteins in the network in an iterative process until a steady-state is attained. Proteins that are relatively close to the seed set get higher propagation scores than distant proteins and are therefore considered to be associated with the phenotype in question. Network propagation is widely used for protein prioritization and related tasks (Cowen et al., 2017).

Formally, given a binary vector *P*_0_ denoting seed proteins, a normalized network adjacency matrix *W* (see below) and a smoothing parameter α controlling the relative importance of the network vs. the seed information, it can be shown that the propagation process converges to a score vector.

$$P=(1-a){\left(I-\alpha W\right)}^{-1}P_{0}$$

Henceforth, we follow (Vanunu et al., 2010) and set α = 0.8 (unless stated otherwise), to allow a fairly high network influence over the prior (seed) knowledge.

There are two main ways by which the adjacency matrix *A* (which could be weighted or unweighted) is normalized to ensure the convergence of the process: (i) a symmetric variant in, which *W* = *D*^−1/2^*AD*^−1/2^ and (ii) a degree-based variant, in which *W* = *AD*^−1^. Here *D* denotes the diagonal weighted degree matrix.

### Previously Suggested Normalization Solutions

The raw scores from the propagation process do not carry a statistical meaning, and highly depend on the size of the seed set and the degrees of the proteins involved. It is thus desirable to normalize them. In the following we describe three previous normalization methods and a new hybrid of two of the methods; full details can be found in the Methods.

Erten et al. (2011) suggested the DADA method that builds on normalizing each propagation score by the eigenvector centrality measure of the same protein, which can be calculated by propagating with *α* = 1 from the same seed set (Brin and Page, 1998; Bryan and Leise, 2006; Erten et al., 2011). Here we analyze both this simple EC method and the full DADA method which uses ranks (rather than the scores themselves) of the regular propagation scores in case the average weighted degree of the seed set exceeds the network average weighted degree, or the logarithm of the EC score otherwise.

Mazza et al. (2016) suggested normalizing propagation scores by comparing them to propagations from random seed sets (RSS). This method produces *p*-values and is implemented as a web tool at http://anat.cs.tau.ac.il/WebPropagate/ (Biran et al., 2018).

We also examine here a hybrid of RSS and DADA, which we call RSS_SD. This variant produces *p*-values in the same manner RSS does, but the random seed sets are chosen to be degree-distributed like the original seed set using the method of Erten et al. (2011).

### Normalization With Random Degree-Preserving Networks (RDPN)

**FIGURE 1:**
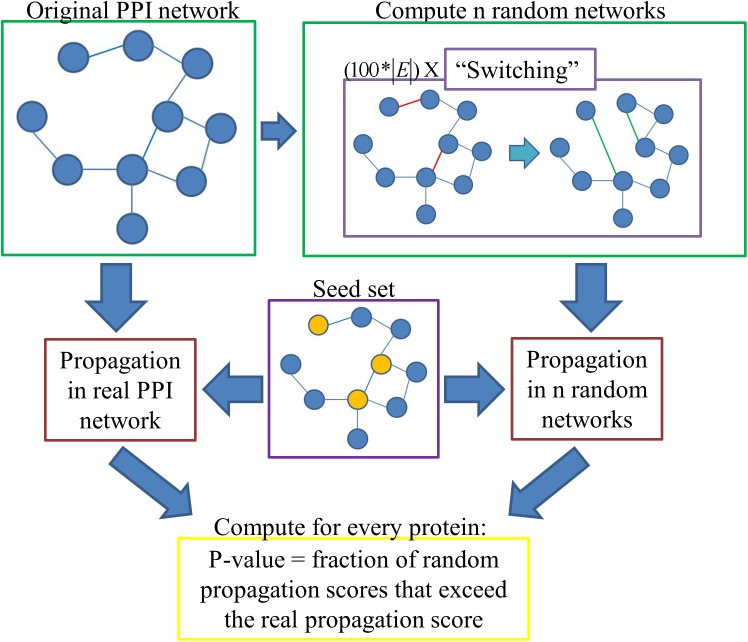
Schematic pipeline of the RDPN method.

The only previous normalization method we are aware of that assigns statistical significance to the propagation scores is based on propagating random seed sets. Such computations do not take into account the degrees of the seed nodes. To overcome this shortcoming, we propose a novel method that is based on randomizations of the input network rather than the seed sets. Specifically, the propagation score of a protein is compared to the scores the protein attains on random degree-preserving networks under the same seed set. Our normalization method with random degree-preserving networks, RDPN, is schematically depicted in Figure 1.

In order to execute this method, one first has to compute *n* random degree-preserving networks (we use *n* = 100 unless otherwise stated). We implemented the “switching” method, in which in each iteration two edges (*u, v*) and (*s, t*) are picked randomly, and if *u*≠*v*≠*s*≠*t* and the edges (*u, t*), (*s, v*) do not already exist, then they are “switched,” namely the edges (*u, v*) and (*s, t*) are removed and the edges (*u, t*) and (*s, v*) are added. For the construction of one random network, we executed 100^∗^|E| such iterations, where |E| denotes the number of edges in the network, per the recommendation in Milo et al. (2003).

One issue that immediately emerges is the question of connectivity. Network propagation relies on the fact that all relevant proteins are part of one connected component, otherwise the information will not diffuse in a desired way. For example, suppose that during the randomization process two proteins got disconnected from the main component, creating a very small connected component of their own. If one of them is a seed protein, then the propagation score of the other one will be unreasonably high. However, if none of them is a seed protein, then their propagation scores will be 0. We addressed this issue by considering for each protein only the instances in which it was part of the main connected component in the network.

In detail, *p*-values are computed as follows: Each protein *v* gets a “real” propagation score $X_{real}^{v}$ by propagating from the seed set on the original network; it also gets *n* random scores $X_{i}^{v}$ (0 ≤*i* ≤*n*-1) by propagating from the same seed set on the *n* random networks. Then its *p*-value is computed as the fraction of random instances in which its score exceeded its real propagation score, i.e.:

$$p^{v}=\frac{|\{i|(X_{i}^{v}\geq X_{real}^{v}\;and\;v\;is\;part\;of\;the\;main\;connected\;component\;in\;the\;i'th\;network)\}|+1}{|\{i|(v\;is\;part\;of\;the\;main\;connected\;component\;in\;the\;i'th\;network)\}|+1}$$

To overcome the infrequent case in which a protein has a high tendency to get disconnected and, therefore, its *p*-value is determined based on an insufficient number of instances, we determined that a protein with less than *n*/2 relevant instances (instances in which it was part of the main connected component) will be assigned a *p*-value of one. Empirically, in our pre-computed random networks there was no such protein and therefore this condition was never used.

### Performance Evaluation

We compared the basic propagation computation with the three previously suggested normalization techniques (EC, DADA, and RSS), RSS_SD and our own Random Degree-Preserving Networks (RDPN) normalization with respect to their performance in multiple disease-related and function-related prioritization tasks as described below.

#### Overall Performance

We evaluated the performance of the six methods and two matrix normalization variants on four large-scale data sets in a fivefold cross validation setting. Each data set contained multiple groups of function-related or disease-related genes with respect to which the prioritization of each normalization method was evaluated. Each method’s performance was summarized by the area under the ROC curve (AUROC) measure, when using similar-degree negative samples (Methods).

The evaluation results are given in Table 1. Regarding the two variants of adjacency matrix normalization, we found that in 12 out of 24 method-data set pairs (and also on average) the symmetric variant performs better (in 10 of them the degree-based variant performed better, and 2 were ties). Therefore, we focused on this variant in all subsequent evaluations. On average, the three top performing normalization methods were RDPN, RSS_SD, and EC, attaining similar AUROCs across the four data sets.

**Table 1:** Average AUROC of the six methods across four data sets, using two variants of adjacency matrix normalization.

Dataset	Symmetric adjacency matrix normalization						Degree-based adjacency matrix normalization					
		
	Propagation	EC	DADA	RSS	RSS_SD	RDPN	Propagation	EC	DADA	RSS	RSS_SD	RDPN
**Menche-OMIM**	0.695	0.74	0.707	0.729	0.745	**0.746**	0.663	**0.742**	0.685	0.738	**0.742**	**0.742**
**GO_MF**	0.76	0.83	0.783	0.805	0.827	**0.832**	0.715	0.83	0.749	0.826	**0.832**	0.831
**GO_CC**	0.763	**0.833**	0.782	0.812	0.829	**0.833**	0.721	**0.833**	0.75	0.83	**0.833**	0.831
**GO_BP**	0.74	0.798	0.757	0.774	0.797	**0.801**	0.707	0.802	0.734	0.798	0.8	**0.803**


*For each dataset, the best performing method in each variant is shown in bold.*

**FIGURE 2:**
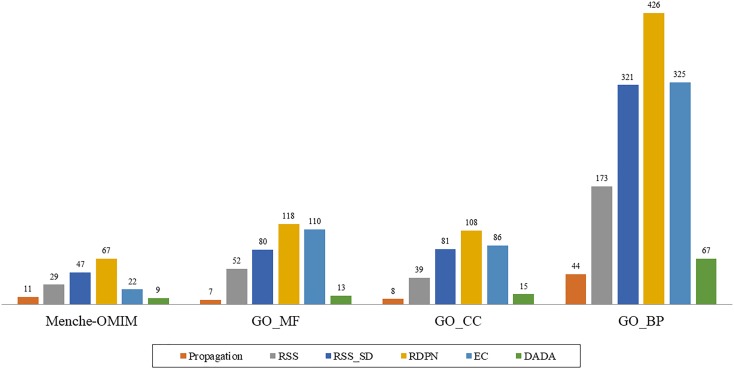
“Best method” counts, based on the AUROC measure, of the six methods across four data sets: Menche-OMIM (173 diseases), GO-MF (358 terms), GO-CC (306 terms), and GO-BP (1237 terms).

However, when examining the performance on the individual groups within the data sets, we found that the RDPN method greatly outperformed all others with the highest number of groups for which it gave the best results across all data sets (Figure 2).

#### Degree Bias of the Different Methods

**FIGURE 3:**
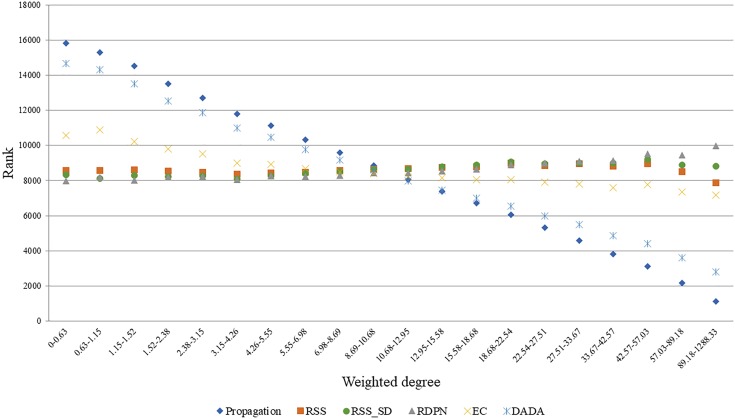
Average rank vs. weighted degree of candidate proteins. Depicted here are ranks based on seed sets from five arbitrary diseases in the Menche-OMIM set (Menche et al., 2015); bins contain approximately equal numbers of proteins. Ranks are derived from the methods’ scores the better the score the lower the rank.

A good normalization method should account for the degrees of the candidate proteins, as these influence propagation scores. To test this, we focused on the Menche-OMIM set. Expectedly, the raw propagation scores are highly correlated with the weighted degree of the candidate protein (0.901 Spearman correlation). A similar anti-correlation level (-0.749) was observed for DADA’s ranks. In contrast, EC scores were only weakly correlated with the candidate protein weighted degree (average Spearman coefficient of 0.238), and the *p*-values computed by RSS, RSS_SD, and RDPN were relatively unbiased (average Spearman coefficients of 0.019, 0.035, and 0.078, respectively). These results are depicted in Figure 3.

#### *P*-Value Biases

**FIGURE 4:**
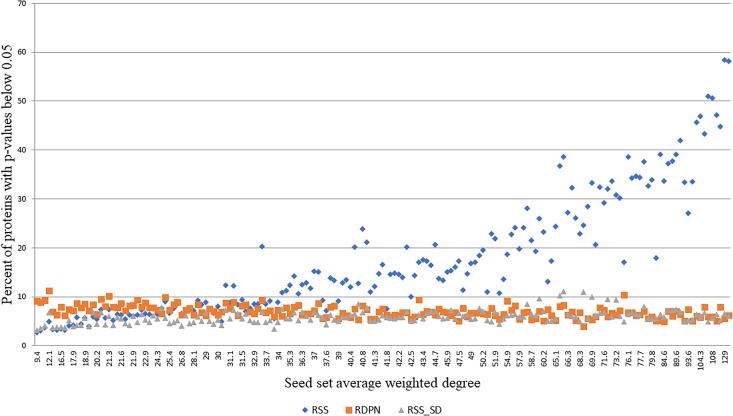
Percent of proteins with *p*-values below 0.05 vs. seed set average weighted degree, using 173 seed sets from the Menche-OMIM data set (Menche et al., 2015).

While the regular propagation, EC and DADA produce scores or ranks, which are only expected to be meaningful for ranking proteins within the same run, RSS, RSS_SD, and RDPN produce *p*-values, which can be thresholded within and across runs to yield statistically significant hits. In order to evaluate the robustness of the assigned *p*-values, we tested their dependence on the average weighted degree of the seed set, focusing on the Menche-OMIM set. We found that both RDPN’s and RSS_SD’s percents of significant hits (*p*-value < 0.05) are only mildly affected by the seed set average weighted degree (Spearman correlation coefficients of -0.511 and 0.427, respectively) and are robust across runs (stds of 1.23 and 1.34%, respectively), while RSS’s percent of significant hits is both strongly correlated with the seed set average weighted degree (Spearman 0.945) and much more sensitive to the input seed set (std 12.46%) (Figure 4).

### A Telomere-Length Maintenance Case Study

In order to study the biological implications of the different normalization methods, we used a telomere length maintenance (TLM) data set from yeast. Specifically, we used a seed set of known TLM genes from Askree et al. (2004) (see Methods and Supplementary Table S1). We compiled lists of top-ranking proteins by looking at the top 30 proteins for each of the methods (for RSS, RSS_SD, and RDPN we used *n* = 5000 to increase the resolution of *p*-values produced). We then manually evaluated the relevance of these predicted proteins to telomere length maintenance based on the literature (Table 2). We found that the basic propagation produced 4 TLM-related proteins (out of 30), EC produced 5, DADA produced 11, RSS produced 10, RSS_SD produced 12 and RDPN produced 25. This high specificity (25/30) highlights again the advantage of the newly suggested normalization over previous ones. The newly identified proteins participate in telomere length maintenance as part of large complexes or pathways, such as the VPS pathway, the THO, Mediator and RPD3 complex. The RDPN procedure correctly identified known proteins of these complex previously not characterized. Moreover, out of the 5 proteins not known to be involved in telomere length maintenance, two of them (RNH202 and RNH203) encode subunits of the Rnase H, a nuclease with important roles in genome maintenance, mutated in the human Aicardi-Goutieres syndrome (Crow et al., 2006). Its roles in R-loop repair have suggested possible involvement in telomere biology, although no clear telomere length defect has been detected (Lafuente-Barquero et al., 2017).

**Table 2:** Top 30 proteins obtained by the different methods in the telomere-length maintenance case study.

	Propagation	EC	DADA	RSS	RSS_SD	RDPN
1	**VPS20^15^**	LIP2	**VPS20^15^**	TFG2	**SAE2^8,13^**	**VPS24^1,10^**
2	SSB1	RNH203	**SRN2^1,10^**	SCW10	**GBP2^7,14^**	**SDS3^5^**
3	SSA1	RPI1	SSA1	RPB3	**TEX1^6^**	**SRN2^1,10^**
4	RPN11	RNH202	SSB1	SUB2	**HRB1^4^**	MGM1
5	HHT1	PMT5	RNH203	**DOA4^12^**	**THO2^6^**	**THO2^6^**
6	**SRN2^1,10^**	**SRN2^1,10^**	RPN11	CPR7	**VPS20^15^**	**RSC8^16^**
7	CRM1	RFU1	RNH202	RPO21	CPR7	**VPS21^15^**
8	HHT2	FLO11	HHT1	**GBP2^7,14^**	PAF1	**VPS20^15^**
9	HHF1	SPL2	CRM1	**RSC8^16^**	SUB2	**GAL11^12^**
10	HSP82	MVB12	MGM1	DLT1	**RAP1^3^**	RPO21
11	CDC28	**VPS20^15^**	HHT2	UBP16	**SRN2^1,10^**	**VPS41^1,10^**
12	RNH203	MGM1	HHF1	SUP35	BUD17	**MED2^2^**
13	RSP5	FMS1	HSP82	**VPS24^1,10^**	OLA1	**GBP2^7,14^**
14	RNH202	NTG2	RSP5	**RAP1^3^**	RIM8	**VPS33^1,10^**
15	SSB2	SAY1	**VPS24^1,10^**	**HRB1^4^**	MTG2	**SRB6^2^**
16	RPO21	SCW10	RPO21	**TEX1^6^**	**RSC8^16^**	**MED7^2^**
17	HHF2	YKR051W	PEP5	HTB1	RPI1	PEP5
18	DSN1	BSC1	**VPS16^1,10^**	**GAL11^12^**	SUP35	**VPS8^1,10^**
19	MGM1	YBR063C	CDC28	HTA2	RSC3	**RXT2^5^**
20	CMR1	**VPS24^1,10^**	SSB2	SCP160	**VPS8^1,10^**	RNH203
21	**VPS24^1,10^**	PUT3	**THO2^6^**	YPK9	**DOA4^12^**	**MED8^2^**
22	RVB1	MLH3	HHF2	HHT2	MVB12	**VPS4^1,10^**
23	RVB2	IBA57	DSN1	NTG2	PEP5	**RGR1^16^**
24	TOM1	CIA2	**VPS33^1,10^**	STH1	ALG3	**VPS16^1,10^**
25	RPC82	MHF1	**VPS41^1,10^**	HHF1	REB1	**DOA4^12^**
26	SSC1	ERD2	CMR1	MRX1	**SIR2^9,11^**	RNH202
27	PEP5	BUD17	**SRB4^2^**	**RGR1^16^**	RSC9	**CTI6^5^**
28	**SRB4^2^**	**CTF8^12^**	**GAL11^12^**	YPR202W	TFG2	**HRB1^4^**
29	HTA2	RIM8	**RGR1^16^**	**SIR4^12^**	YJL070C	**RAP1^3^**
30	MMS22	**VPS38^1,10^**	**MED8^2^**	SRB4	SCW10	**TEX1^6^**


*Proteins in green are related to the TLM mechanism by the following explanations or references: ^*1*^TLM, belongs to the VPS pathway; ^*2*^part of the mediator complex (with SRB2, SRB3, SRB8, SSN2, SSN3, SSN8, GAL11, MED1, NUT1, PGD1, RGR1, and all TLMs); ^*3*^this is the main telomere-length determining protein; ^*4*^paralog of GBP2, the telomere-binding protein; ^*5*^part of RPD3 complex, as DEP1, SAP30, and SIN3 (TLMs); ^*6*^part of the THO/TREX complex (with THP2, HPR1, MFT1 and SOH1, and all TLMs); ^*7*^telomere binding protein; ^*8*^regulator of the MRX complex that processes telomeres; ^*9*^affects telomere chromatin, although not telomere length; ^*10*^Dieckmann et al. (2016); ^*11*^Ellahi et al. (2015); ^*12*^Gatbonton et al. (2006); ^*13*^Hardy et al. (2014); ^*14*^Konkel et al. (1995); ^*15*^Shachar et al. (2008); ^*16*^Ungar et al. (2009).*

## Conclusion

In summary, we have devised a new method (RDPN) for normalizing propagation results that accounts for the degrees of the involved proteins and produces robust *p*-value estimations. The method was shown to outperform previous ones across diverse disease-related and function-related data sets. Importantly, we have shown that the *p*-values it assigns do not depend on the degree of the protein being scored, hence this method is less prone to literature biases and more likely to discover new associations. Moreover, we have shown that its assigned *p*-values are robust to the average degree of the seed set, allowing significance assessment across different data sets. Finally, in testing the biological implications of the method’s predictions, we found that it greatly outperforms previous normalizations and leads to new biological insights.

Considering all evaluated parameters, it seems that three of the tested methods outshine the others: RDPN, which generates robust *p*-values and displays the best performance, RSS_SD which also generates robust *p*-values but doesn’t perform as well, and EC which is easy to implement and has good performance although its nominal scores are harder to interpret.

We note that there are many variants in the literature of the basic network propagation methodology, such as random walk with restart and diffusion kernel (Cowen et al., 2017). Our normalization method is readily applicable to all these variants and can be used to eliminate potential degree biases and assign statistical significance values.

## Methods

### Normalization Methods

#### Normalization With Random Seed Sets (RSS)

This method uses propagation scores from *n* random seed sets (we use *n* = 100 unless stated otherwise) to normalize the real propagation scores, as suggested by Mazza et al. (2016). In detail, each protein *v* has a “real” propagation score $X_{real}^{v}$ the score it got by propagating from the real seed set; and *n* random scores $X_{i}^{v}$ (0 ≤ *i* ≤*n*-1) derived by propagating from *n* random seed sets (each with the same number of proteins as the real seed set). For every protein *v* only the instances in which it was not part of the random seed set are considered, and its *p*-value is the fraction of random instances in which its score exceeded its real propagation score, i.e.:

$$p^{v}=\frac{|\{i|(X_{i}^{v}\geq X_{real}^{v}\;and\;v\;was\;not\;part\;of\;the\;i'th\;random\;seed\;set)\}|+1}{|\{i|(v\;was\;not\;part\;of\;the\;i'th\;random\;seed\;set)\}|+1}$$

#### Normalization With Eigenvector Centrality (EC)

The EC scores are computed as follows:

$$p^{v}=\frac{X_{\alpha =0.8}^{v}}{X_{\alpha =1}^{v}}$$

where $X_{\alpha =0.8}^{v}$ is the propagation score of protein *v* when propagating from the seed set with *α* = 0.8, and $X_{\alpha =1}^{v}$ is its propagation score when propagating from the same seed set with *α* = 1 (i.e., disregarding the seed set in the computation).

#### DADA

The DADA ranks, as described in Erten et al. (2011), are computed as follows: first EC scores are computed as:

$$EC^{v}=log\left(\frac{X_{\alpha =0.7}^{v}}{X_{\alpha =1}^{v}}\right)$$

for all the proteins in the network where $X_{\alpha =0.7}^{v}$ is the propagation score of protein *v* when propagating from the seed set with α = 0.7, and $X_{\alpha =1}^{v}$ is its propagation score when propagating from the same seed set with α = 1. Then each protein gets a rank $R_{\mathrm{EC}}^{i}$ which is its position in a descending order of EC scores, and also a rank $R_{\mathrm{prop}}^{v}$ which is its position in a descending order of the regular propagation scores $X_{\alpha =0.7}^{v}$. Finally, if the average weighted degree of the seed set exceeds the network average weighted degree, all proteins final ranks are set to $R_{\mathrm{prop}}^{v}$. Otherwise, they are set to $R_{\mathrm{EC}}^{v}$.

#### Normalization With Random Similar Degree Distributed Seed Sets (RSS_SD)

Following Erten et al. (2011), we first construct seed sets *S*(*i*) (0 ≤*i* ≤ *n*-1, we use *n* = 100) that have a degree distribution that is similar to the original seed set *S* by applying this procedure: We assign each *v*∈*V* to a bucket *B*(*u*) such that *u*∈*S* and |*W*(*v*)-*W*(*u*)| is minimized (ties are broken randomly).

In case there are two or more seed proteins with an equal weighted degree, there is a possibility that one of their buckets will remain empty. If that happens, we reassign all network proteins (we repeat this step if necessary).

We generate *S*(*i*) by choosing a protein from each bucket uniformly at random.

We then propagate from these seed sets, as well as from the original seed set, and proceed to compute *p*-values as in the RSS method.

### Data Sets

#### Menche-OMIM Data Set

Menche et al. (2015) compiled a list of 299 diseases defined by the Medical Subject Headings (MeSH) that have at least 20 associated genes from either the Online Mendelian Inheritance in Man (OMIM) data set or the genome-wide association study (GWAS) data set (or both). We empirically found that all methods perform better when using only the genes from OMIM, so only the 173 diseases out of that list that have at least 20 and up to 1000 associated genes from OMIM in the HIPPIE network were used for evaluation.

#### GO Data Set

We used geneSCF (Subhash and Kanduri, 2016) to get a list of all GO terms (Ashburner et al., 2000; The Gene Ontology Consortium, 2017) (in all three sub-ontologies) with their corresponding genes. We focused the evaluation on terms that included between 20 and 1000 genes (1237 GO Biological Process (BP) terms, 306 GO Cellular Component (CC) terms and 358 GO Molecular Function (MF) terms).

#### TLM Data Set

A genome wide-screen study by Askree et al. (2004) found 173 *S. cerevisiae* genes that affect telomere length. We used 163 of them that are found in the ANAT *S. cerevisiae* network as the seed set (Supplementary Table S1).

### PPI Networks

For the performance evaluation section we used the HIPPIE network which has 17335 proteins and 330028 (non self-loops) interactions in its main connected component (Alanis-Lobato et al., 2017) (version 18-Jul-2017).

For the TLM case study we used the ANAT *Saccharomyces cerevisiae* network which has 5527 proteins and 75678 (non self-loops) interactions in its main connected component (Almozlino et al., 2017).

### Area Under ROC Curve (AUROC) Measure

For each group of disease-related or function-related genes, we randomly split it to five equally sized parts. In each cross-validation iteration we hid one of the parts, used the other four as a seed set, and tested the success of the method in predicting the hidden proteins (serving as positive samples) using the AUROC measure. We then averaged the performance across the five iterations. To compute the AUROC scores, we picked negative samples with similar weighted degrees as the positive samples. This was implemented as follows: for each positive protein with a weighted degree *w*, we chose the smallest integer *r* such that there are at least 100 proteins in the network (excluding the seed set, the positive samples and the already chosen negative samples) with weighted degree in the range *[w-r, w+r]*. We then randomly picked a protein from this group to be used as a negative sample.

## Author Contributions

HB and RS conceived the RDPN method and designed the computational framework. HB implemented the framework and produced the results. All authors interpreted the results and contributed to the manuscript.

## Conflict of Interest Statement

The authors declare that the research was conducted in the absence of any commercial or financial relationships that could be construed as a potential conflict of interest. The reviewer MK declared a past collaboration with one of the authors RS.
