# Supplementary material for: Comparative Analysis of Normalization Methods for Network Propagation
Source: Front Genet. 2019 Jan 22;10:4. doi: 10.3389/fgene.2019.00004 (PMC6350446; doi:10.3389/fgene.2019.00004)
Supplement: Supplementary file 1 [file Table_1.docx]

***Supplementary Material***

**Comparative analysis of normalization methods for network propagation**

**Hadas Biran, Martin Kupiec and Roded Sharan^*^**

*** Correspondence:** Prof. Roded Sharan: roded@tau.ac.il

# 1 Supplementary Tables

**Table S1.** Seed set used for the TLM case study.

|  | **Name** | **GeneID** |
| --- | --- | --- |
| **1** | DCC1 | 850344 |
| **2** | VPS23 | 850349 |
| **3** | MAK31 | 850383 |
| **4** | HTL1 | 850384 |
| **5** | RPS14A | 850397 |
| **6** | PHO87 | 850403 |
| **7** | BUD23 | 850414 |
| **8** | HCM1 | 850429 |
| **9** | CSM1 | 850447 |
| **10** | ERJ5 | 850602 |
| **11** | ISA1 | 850632 |
| **12** | HSP104 | 850633 |
| **13** | MMM1 | 850654 |
| **14** | BRE2 | 850702 |
| **15** | VPS32 | 850712 |
| **16** | XDJ1 | 850779 |
| **17** | VPS18 | 850840 |
| **18** | EST1 | 850934 |
| **19** | VPS34 | 850941 |
| **20** | ARV1 | 850943 |
| **21** | EST2 | 851028 |
| **22** | RSC2 | 851071 |
| **23** | SUR4 | 851087 |
| **24** | CCW14 | 851107 |
| **25** | VPS36 | 851135 |
| **26** | CDC73 | 851136 |
| **27** | RIF2 | 851174 |
| **28** | TPD3 | 851217 |
| **29** | DEP1 | 851220 |
| **30** | GPB2 | 851243 |
| **31** | GCV3 | 851254 |
| **32** | NUP60 | 851263 |
| **33** | RPP1A | 851478 |
| **34** | VPS39 | 851482 |
| **35** | SIT4 | 851513 |
| **36** | RPN4 | 851542 |
| **37** | PTC1 | 851558 |
| **38** | RRP8 | 851656 |
| **39** | YDR115W | 851692 |
| **40** | HPR1 | 851716 |
| **41** | HMO1 | 851754 |
| **42** | HDA2 | 851889 |
| **43** | SUM1 | 851905 |
| **44** | XRS2 | 851975 |
| **45** | RPL12B | 852026 |
| **46** | UGO1 | 852081 |
| **47** | VPS3 | 852106 |
| **48** | KRE28 | 852145 |
| **49** | TEL1 | 852190 |
| **50** | LDB7 | 852277 |
| **51** | PDX3 | 852323 |
| **52** | VPS15 | 852394 |
| **53** | AGP2 | 852429 |
| **54** | RIF1 | 852578 |
| **55** | APE3 | 852589 |
| **56** | RTF1 | 852607 |
| **57** | VPS43 | 852660 |
| **58** | KEM1 | 852702 |
| **59** | HUR1 | 852708 |
| **60** | NUT1 | 852726 |
| **61** | RPL1B | 852742 |
| **62** | SOH1 | 852750 |
| **63** | GUP1 | 852796 |
| **64** | RPB9 | 852810 |
| **65** | YGL039W | 852844 |
| **66** | CDH1 | 852881 |
| **67** | CAX4 | 852924 |
| **68** | YGR042W | 852933 |
| **69** | LST7 | 852948 |
| **70** | UPF3 | 852963 |
| **71** | PCP1 | 852993 |
| **72** | SRB5 | 852996 |
| **73** | SMI1 | 853144 |
| **74** | YTA7 | 853186 |
| **75** | YOR1 | 853198 |
| **76** | RPB4, CTF15 | 853301 |
| **77** | TRK1 | 853312 |
| **78** | ARG2 | 853374 |
| **79** | POL32 | 853500 |
| **80** | HIT1 | 853516 |
| **81** | FMP26 | 853543 |
| **82** | VPS25 | 853566 |
| **83** | ADO1 | 853569 |
| **84** | EAP1 | 853631 |
| **85** | MRPL38 | 853684 |
| **86** | CTK1 | 853718 |
| **87** | MRT4 | 853860 |
| **88** | YOL138C | 853982 |
| **89** | HST1 | 854086 |
| **90** | TAT2 | 854139 |
| **91** | SIN3 | 854158 |
| **92** | PHO80 | 854161 |
| **93** | YSP3 | 854164 |
| **94** | ELG1 | 854315 |
| **95** | NFI1 | 854327 |
| **96** | MET7 | 854415 |
| **97** | RFM1 | 854453 |
| **98** | RPS10A | 854468 |
| **99** | PMT3 | 854499 |
| **100** | YOR322C | 854500 |
| **101** | MMS19 | 854678 |
| **102** | YIL042C | 854769 |
| **103** | CST6 | 854775 |
| **104** | EST3 | 854806 |
| **105** | VPS9 | 854876 |
| **106** | GTR1 | 854918 |
| **107** | MFT1 | 854940 |
| **108** | PIF1 | 854941 |
| **109** | OGG1 | 854942 |
| **110** | YML035C | 854973 |
| **111** | RPS17A | 854984 |
| **112** | MOT3 | 855092 |
| **113** | UPF1 | 855104 |
| **114** | NPL6 | 855116 |
| **115** | YKU80 | 855132 |
| **116** | ASC1 | 855143 |
| **117** | STO1 | 855155 |
| **118** | RPL13B | 855173 |
| **119** | RPS16A | 855174 |
| **120** | MLH1 | 855203 |
| **121** | SPT21 | 855217 |
| **122** | HSC82 | 855224 |
| **123** | MRE11 | 855264 |
| **124** | MRPL44 | 855265 |
| **125** | SAP30 | 855305 |
| **126** | YMR269W | 855311 |
| **127** | YKU70 | 855328 |
| **128** | HCH1 | 855440 |
| **129** | RAD50 | 855471 |
| **130** | VPS75 | 855475 |
| **131** | URE2 | 855492 |
| **132** | ADE12 BRA9 | 855501 |
| **133** | RNH35 | 855652 |
| **134** | YDJ1 | 855661 |
| **135** | SSN8 | 855706 |
| **136** | LEA1 | 855888 |
| **137** | GUP2 | 855912 |
| **138** | BEM4 | 855942 |
| **139** | YPL144W | 855959 |
| **140** | SSE1 | 855998 |
| **141** | YPL105C | 855999 |
| **142** | BRO1 | 856021 |
| **143** | VPS28 | 856040 |
| **144** | YPL041C | 856066 |
| **145** | PHO85 | 856076 |
| **146** | VPS22 | 856105 |
| **147** | CSR2 | 856142 |
| **148** | ATG11 | 856162 |
| **149** | MAK3 | 856163 |
| **150** | TOM5 | 856252 |
| **151** | OPI1 | 856366 |
| **152** | PRS3 | 856375 |
| **153** | SRB2 | 856437 |
| **154** | PPE1 | 856474 |
| **155** | UPF2 | 856476 |
| **156** | LRP1 | 856481 |
| **157** | SPS100 | 856541 |
| **158** | THP2 | 856572 |
| **159** | RPS4B | 856610 |
| **160** | YEL057C | 856653 |
| **161** | MAK10 | 856657 |
| **162** | BUD16 | 856683 |
| **163** | BEM2 | 856899 |
